# Serial postoperative peritoneal fluid analyses in horses with naturally‐occurring strangulating and non‐strangulating gastrointestinal lesions

**DOI:** 10.1111/vsu.70089

**Published:** 2026-02-22

**Authors:** Maria E. Granello, Jenna M. Young, Dana B. Cleff, Emma B. M. Banks, Troy N. Trumble

**Affiliations:** ^1^ Department of Veterinary Population Medicine, College of Veterinary Medicine University of Minnesota Saint Paul Minnesota USA

## Abstract

**Objective:**

To describe characteristics of postoperative peritoneal fluid following exploratory laparotomy for naturally‐occurring gastrointestinal lesions in horses.

**Study design:**

Prospective, observational cohort study.

**Animals:**

A total of 26 client‐owned horses that underwent exploratory laparotomy for naturally‐occurring gastrointestinal lesions.

**Methods:**

Abdominocentesis was performed pre‐ or intraoperatively, and at three time points postoperatively (24, 72, and 168 h). Peritoneal lactate, total protein (TP), total nucleated cell count (TNCC), cytology, and systemic lactate were performed at each time point, if possible. To account for repeated measures, a linear mixed model analysis was performed for each dependent variable listed above.

**Results:**

Horses were divided into groups based on the gastrointestinal lesion diagnosed at surgery (14 strangulating and 12 non‐strangulating). Peritoneal lactate (*p* < .001) and TP (*p* = .02) were significantly higher preoperatively in horses with strangulating compared to non‐strangulating lesions, with no significant differences between lesion groups for any postoperative measurement. Peritoneal lactate and TP concentrations remained above normal for the entire postoperative study period in both groups. Systemic lactate returned to normal concentrations by 24 h postoperatively with both groups being significantly lower than preoperative concentrations (*p* = .02). Peritoneal TNCC concentrations increased in strangulating (*p* = .001) and non‐strangulating (*p* < .001) horses at 24 h postoperatively compared to preoperatively.

**Conclusion:**

Regardless of lesion, peritoneal fluid lactate and TP remained above normal at 1 week following exploratory laparotomy for naturally‐occurring gastrointestinal lesions in horses.

**Clinical significance:**

Current reference values for preoperative fluid sample analyses should not be used in the postoperative period.

## 1 INTRODUCTION

Colic is a common cause of morbidity and mortality in horses,^1^ and can be caused by a wide range of disease processes, with many being self‐limiting. However, some horses require surgery for strangulating or non‐strangulating gastrointestinal lesions. Many diagnostic techniques are available to determine if a horse requires surgery, including collection and evaluation of peritoneal fluid.^2^, ^3^, ^4^, ^5^, ^6^, ^7^, ^8^ One specific component of peritoneal fluid is lactate, a byproduct of anaerobic cellular metabolism.^3^ Lactate concentrations can elevate in peritoneal fluid when there is compromised circulation or ischemia present.^3^ Along with measurement in systemic blood, peritoneal lactate has been used to determine if a strangulating lesion is present that would require surgical intervention.^3^, ^4^, ^5^, ^8^, ^9^, ^10^

Recurring colic pain is the most common short‐term complication following exploratory laparotomy in horses, with 28% of horses experiencing postoperative colic pain.^11^ Diagnostic procedures in horses experiencing colic in the postoperative period are similar to those used preoperatively. However, the effects of surgery on the peritoneal fluid in the postoperative period are relatively unknown, making it difficult to utilize this diagnostic tool to determine if another surgery is required. Characteristics of equine peritoneal fluid, including total nucleated cell count (TNCC) and total protein (TP), have been previously reported postoperatively in healthy horses undergoing abdominal surgery.^12^, ^13^, ^14^ However, peritoneal lactate was not examined in these studies. More importantly, there is a lack of research describing the characteristics of equine peritoneal fluid postoperatively in horses with naturally‐occurring gastrointestinal disease. Due to this lack of information in horses with naturally‐occurring disease, the utility of peritoneal fluid analysis is limited in these challenging postoperative colic cases. Understanding the changes that occur in peritoneal fluid after surgical intervention for gastrointestinal disease would provide clinicians with another possible diagnostic and/or prognostic tool as they manage cases of postoperative colic and determine the need for repeat laparotomy.

Our objective in this study was to report pilot data for peritoneal fluid in the postoperative period in horses following surgical correction of naturally‐occurring gastrointestinal lesions. Specifically, we aimed to measure the lactate, TP, and TNCC in these cases. We hypothesized that peritoneal fluid lactate and TP would decrease in the postoperative period, that TNCC would decrease after 24 h postoperatively but not return to preoperative levels within 7 days, and that systemic lactate would decrease postoperatively and return to normal reference range more rapidly than peritoneal fluid lactate.

## 2 MATERIALS AND METHODS

### 2.1 Study subjects

A prospective, observational cohort study was designed. Horses, which presented to the University of Minnesota for colic from July 1, 2023 to June 1, 2025, were candidates for enrollment. Horses underwent a physical examination at the time of presentation, and further diagnostics were performed according to the managing clinician's preference, including nasogastric intubation, rectal palpation, abdominal ultrasound, systemic bloodwork, and/or abdominocentesis. Horses that underwent exploratory laparotomy for acute gastrointestinal colic and recovered from anesthesia were eligible for enrollment. If the horse was euthanized intraoperatively, it was excluded from the study, as were horses under 2 years of age. After the horse had recovered from surgery, informed consent was obtained from owners prior to enrollment in the study. Horses were treated according to the preference of the managing clinician in the postoperative period. The study was approved by the Institutional Animal Care and Use Committee (IACUC) at the University of Minnesota.

### 2.2 Procedures

#### 2.2.1 Data and sample collection protocol

After enrollment, the following data were gathered from the medical record and recorded: age, weight, sex, breed, intake vital parameters, mucous membrane color and capillary refill time, presence of gastrointestinal sounds, rectal examination findings, abdominal ultrasound findings, net reflux retrieved, if preoperative trocharization was performed, and abdominocentesis findings (lactate, TP, TNCC, and cytology). Because diagnostics were performed based on the preference of the attending clinician, not all horses had a complete set of data. Systemic PCV, TP, and CBC findings were recorded for all horses. If a preoperative abdominocentesis was not performed due to clinician discretion, peritoneal fluid was collected immediately after opening the peritoneum during exploratory laparotomy. An extension set attached to a 12 mL syringe was introduced into the abdomen by hand and directed towards the inguinal region, and a sample of peritoneal fluid was collected. If the initial sample was grossly blood‐contaminated, a second sample was collected in the same fashion.

Intraoperative surgical findings were recorded, including the presence or absence of a strangulating lesion, and were classified by the attending board‐certified veterinary surgeon. Strangulating lesions were defined as lesions to any portion of the gastrointestinal tract that occluded or obstructed venous and/or arterial blood supply. Non‐strangulating lesions were defined as gastrointestinal lesions that did not cause vascular occlusion. Further surgical procedures were recorded, including whether a resection and anastomosis was performed, an enterotomy was performed, or carboxymethylcellulose was used. Abdominal lavage was not routinely performed prior to incisional closure. Horses were then divided into strangulating or non‐strangulating cohorts depending upon their surgical lesions.

For all enrolled horses, serial abdominocenteses were collected at 24, 72, and 168 h postoperatively to evaluate the characteristics of peritoneal fluid in the immediate postoperative period. Fluid was collected within ±2 h of the time points listed to accommodate patients' treatment schedules. If a horse was discharged prior to 168 h postoperatively, a sample was collected within 6 h of discharge. If a patient required a second surgical intervention, or euthanasia, during the study period, a sample was collected upon opening the abdomen during the second laparotomy, or immediately following euthanasia if the owner declined a repeat laparotomy for the horse, using the same technique described below.

For each postoperative time point, venous blood was collected via jugular vein using a 20 gauge needle. To allow for collection of postoperative abdominocenteses, horses were sedated with 0.01 mg/kg IV detomidine hydrochloride (Dormosedan, Zoetis, Parsippany, New Jersey). If adequate sedation was not achieved, an additional 0.005–0.01 mg/kg detomidine hydrochloride was given IV. If abdominal fluid could not be collected after two doses of sedation were administered, the procedure was terminated for that time point. The right side of the cranial ventral abdomen had been clipped previously in preparation for colic surgery, and was aseptically prepared using chlorhexidine and isopropyl alcohol. Lidocaine hydrochloride 2% (MWI, Boise, Idaho) was infiltrated locally (4–6 mL), caudal to the xiphoid process, and to the right of ventral midline to provide local anesthesia to the skin, subcutaneous tissues, and underlying muscle. The area was once again aseptically prepared. A #15 blade was used to create a stab incision through the anesthetized region of skin and external abdominal oblique fascia. A teat cannula was then introduced into the abdomen to collect peritoneal fluid via gravity flow. If fluid did not readily drip from the cannula, a sterile 6 mL syringe was attached to assist fluid collection. If fluid collection with the cannula was unsuccessful, up to four 18 gauge 1.5‐inch needles were inserted into the abdomen in slightly varied positions within the aseptically prepared region of skin. Generally, these locations were either cranial to the initial abdominocentesis, but still caudal to the xiphoid process, or slightly abaxial of the initial abdominocentesis site. Again, gravity flow was used to collect peritoneal fluid and a sterile 6 mL syringe was attached to assist in collection, if needed. Ultrasonographic guidance was not routinely used during abdominocentesis. If an enterocentesis was performed, the owner was contacted and the horse was monitored as described in the approved IACUC.

#### 2.2.2 Sample handling and analysis

A PCV, TP (Palm Abbe, Misco, Solon, Ohio), and lactate (Lactate Plus Meter, Nova Biomedical, Waltham, Massachusetts) were measured immediately on blood using stall‐side instrumentation at all postoperative time points. The lactometer has previously been validated for use on equine blood and peritoneal fluid.^15^ At the 72‐ and 168‐h time points, blood was stored in evacuated glass tubes containing EDTA and a complete blood count (Advia 2120, Siemans Medical Solutions USA, Malvern, Pennsylvania) was reviewed by a board‐certified veterinary pathologist. The peritoneal to systemic lactate ratio was calculated by dividing the peritoneal lactate concentration by the systemic lactate concentration, and the peritoneal to systemic lactate difference was calculated by subtracting the systemic lactate from the peritoneal lactate concentration.

On all abdominocenteses, the sample was collected in an evacuated glass tube containing EDTA, and lactate and TP were immediately measured, as described above. The sample was then submitted for body fluid analysis, which included TNCC, description of color, and cytology, by a board‐certified veterinary pathologist. If there was insufficient volume for all testing to be completed, lactate and TP were prioritized. If there was greater than 0.5 mL of fluid remaining, a body fluid analysis was submitted, and if less than 0.5 mL of fluid was remaining, only a cytology was performed. Cytology included a 100‐ or 200‐differential cell count, unless cellularity was too low to perform this count.

Based on previously published reference intervals and values used by attending clinicians in our hospital, normal values for both systemic and peritoneal lactate were defined as ≤1.5 mmol/L for the current study.^3^, ^4^, ^16^ Normal peritoneal total protein was defined as <2.0 g/dL.^4^, ^6^, ^13^, ^17^ Normal peritoneal TNCC was defined as <5000 cells/mL, and normal neutrophil percentage in peritoneal fluid was defined as up to 80% neutrophils (range: 40%–80%).^4^, ^6^, ^13^, ^17^

#### 2.2.3 Follow‐up

An owner questionnaire regarding complications following discharge was conducted via email or telephone a minimum of 5 months after discharge from the hospital. If the horse was examined by a veterinarian at the University of Minnesota after discharge, the medical record was reviewed.

### 2.3 Statistical analysis

Normality was assessed for each dependent variable by analyzing the Shapiro–Wilk test, histograms, and Q‐Q plots for each group (strangulating and non‐strangulating) and over time (hours 0 [preoperative], 24, 72, and 168) using SPSS (version 29.0.2.0). Based on these analyses, data transformations were performed on the following dependent variables prior to further analyses: log transformation for peritoneal lactate, systemic lactate, peritoneal to systemic lactate ratio, and peritoneal TNCC; cube root transformation for peritoneal to systemic lactate difference. Total protein and percent neutrophil data were not transformed. If data were log transformed for analyses, they were back transformed into geometric means (equivalent to the median for log‐normal distribution) for reporting in the results. Due to missing data, a linear mixed model analysis was performed for each dependent variable listed above to account for repeated measures. Fixed effects were defined as the lesion group (strangulating or non‐strangulating), time (hours 0 [preoperative], 24, 72, and 168), and their interaction, with the horse considered a random effect. The Geisser–Greenhouse correction was performed due to non‐sphericity of the data. Tukey's multiple pairwise comparison tests were performed on all dependent variables, except TNCC, to determine differences between groups, and over time. The TNCC had too many missing data points at 72 and 168 h, so only preoperative and 24 h samples were analyzed in the mixed model; an uncorrected Fisher's LSD was then used for multiple comparisons. A Spearman r correlation was performed between all peritoneal and systemic lactate concentrations, as well as between all TNCC and peritoneal lactate and TP concentrations. *p*‐values < .05 were considered statistically significant. Mixed model and correlation analyses were performed using GraphPad Prism (version 10.3.0). Descriptive statistics were performed as indicated.

## 3 RESULTS

During the study period, 50 horses were eligible for enrollment into the study of which 26 horses were enrolled (Table S1), with owners declining enrollment for the remaining. A total of 15 breeds were represented, including four mares and 22 geldings. The mean (±SD) age was 15 ± 5.5 years, and weight was 485 ± 141 kg. The initial sample was collected preoperatively in 9/26 horses and intraoperatively in 17/26 horses. A total of 14 horses were diagnosed with a strangulating lesion, of which 12 had lesions primarily affecting the small intestine and two had lesions affecting the large intestine. A total of 12 horses were diagnosed with a non‐strangulating lesion, of which nine had lesions primarily affecting the large intestine or cecum and three had lesions affecting the small colon (Table S1). An enterotomy alone was performed in 12/26 (46%) horses. The majority of enterotomies were performed in the pelvic flexure of the large colon, but one typhlotomy and one jejunal enterotomy were also performed. A resection alone was performed in 7/26 (27%) horses with another 3/26 (12%) horses having a resection and an enterotomy performed; all resections involved strangulating lesions of the small intestine. Only 4/26 (15%) horses did not have any segment of bowel opened during the laparotomy. Carboxymethylcellulose was used intraoperatively in 20/26 (77%) surgeries, and abdominal lavage prior to closure was performed in 3/26 (12%) surgeries (Table S1).

**FIGURE 1:**
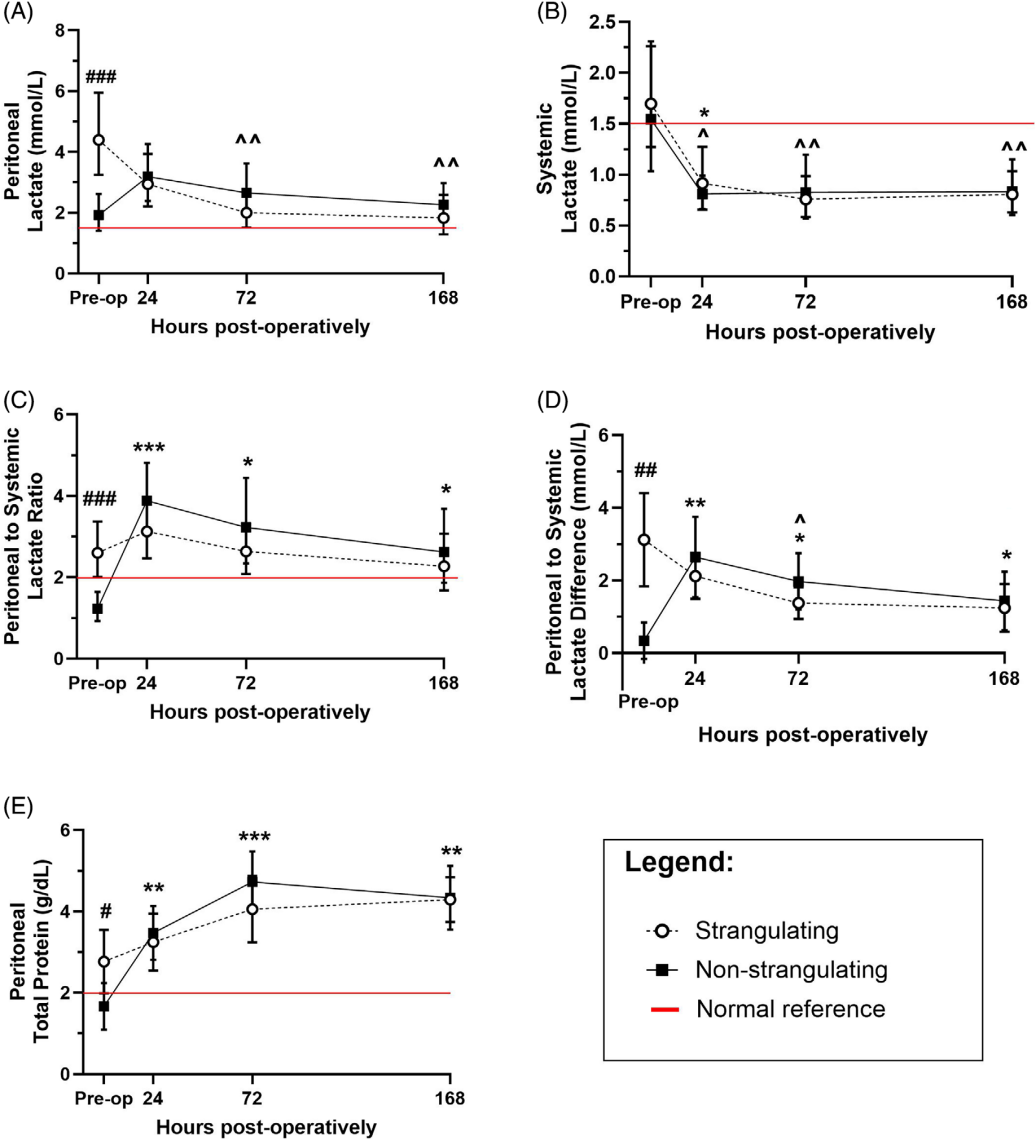
For graphs A, B, and C the geometric mean (approximate median) ± lower and upper 95% CI, and for graphs D and E the mean ± 95% CI lower and upper 95% CI of each time point is represented for horses with naturally‐occurring strangulating (circles; dashed line) or non‐strangulating (squares; solid line) gastrointestinal lesions assessed preoperatively (preop) and at 24, 72, and 168 h postoperatively following exploratory laparotomy. Red lines indicate the relevant high end of the reference interval for a given measurement/calculation. (A) Peritoneal lactate (mmol/L), with the red line at 1.5 mmol/L. (B) Systemic lactate (mmol/L), with the red line at 1.5 mmol/L. (C) Ratio of peritoneal to systemic lactate, with the red line at a ratio of 2. (D) Difference between peritoneal and systemic lactate (mmol/L). (E) Peritoneal total protein (g/dL), with the red line at 2 g/dL. Statistical differences are represented by the following symbols: # represents the difference between strangulating and non‐strangulating cohorts at a given time point; ^ represents the difference of a postoperative time point from preoperative values in the strangulating cohort; and * represents the difference of a postoperative time point from preoperative values in the non‐strangulating cohort. Any three symbols together represent a significant difference ≤ .001, any two symbols ≤ .01, and any single symbol ≤ .05.

For peritoneal lactate, the mixed model analysis revealed a significant main effect for time (F [2.5, 54.4] = 5.1, *p* = .005) and the interaction between time and lesion (F [3, 65] = 11.7, *p* < .001). Post hoc pairwise comparisons demonstrated that preoperative lactate was significantly higher (*p* < .001) in strangulating (geometric mean [lower 95% CI‐upper 95% CI]; 4.40 mmol/L [3.25–5.95]) compared to non‐strangulating (1.92 mmol/L [1.41–2.62]) lesions (Figure 1A; Tables S2 and S5). Horses with strangulating lesions had significantly decreased peritoneal lactate concentrations over time, with postoperative concentrations at 72 h (2.00 mmol/L [1.52–2.65], *p* = .002) and 168 h (1.90 mmol/L [1.29–2.59], *p* = .003) being significantly lower than preoperative concentrations. No significant differences were present in horses with non‐strangulating lesions compared to preoperative concentrations, or in postoperative concentrations between horses with strangulating and non‐strangulating lesions (Table S5). Only six horses had a peritoneal lactate concentration in the normal range by 168 h postoperatively.

For systemic lactate, the mixed model analysis revealed a significant main effect for time (F [2.2, 49.5] = 16.4, *p* < .001) only. Post hoc pairwise comparisons demonstrated that horses with strangulating lesions had significantly lower postoperative concentrations at 24 h (geometric mean [lower 95% CI‐upper 95% CI]; 0.91 mmol/L [0.66–1.27], *p* = .02), 72 h (0.76 mmol/L [0.58–0.99], *p* = .002), and 168 h (0.83 mmol/L [0.63–1.04], *p* = .005) compared to preoperative (1.69 mmol/L [1.27–2.26]) concentrations. In addition, non‐strangulating lesions also had significantly lower postoperative concentrations at 24 h (0.81 mmol/L [0.66–0.99], *p* = .02) compared to preoperative (1.55 mmol/L [1.03–2.31]) concentrations (Figure 1B; Tables S3 and S5). No significant differences were present between horses with strangulating and non‐strangulating lesions.

For the peritoneal to systemic lactate ratio (Figure 1C), the mixed model analyses revealed a significant main effect for time (F [2.7, 57.5] = 11.9, *p* < .001) and the interaction between time and lesion (F [3, 64) = 8.3, *p* < .001). Post hoc pairwise comparisons demonstrated that preoperative peritoneal to systemic lactate ratio had significantly higher values in horses with strangulating (geometric mean [lower 95% CI‐upper 95% CI]; 2.60 [2.01–3.37]) compared to non‐strangulating lesions (1.23 [0.93–1.64], *p* < .001; Table S5). Horses with non‐strangulating lesions had significantly higher postoperative values at 24 h (3.89 [3.13–4.81], *p* < .001), 72 h (3.22 [2.34–4.44], *p* = .01), and 168 h (2.63 [1.87–3.68], *p* = .03) compared to preoperative values for the ratio. No significant differences were present postoperatively between horses with strangulating and non‐strangulating lesions (Table S5). At 24 h postoperatively, systemic lactate returned to normal in 23/26 horses while peritoneal lactate remained elevated in 23/24 horses with available samples. The concentrations significantly correlated (*p* < .001) between systemic and peritoneal lactate with *R* = .504.

For the peritoneal to systemic lactate difference (Figure 1D), the mixed model analyses revealed a significant main effect for time (F [1.9, 40.5] = 6.2, *p* = .005) and the interaction between time and lesion (F [3, 64] = 17.5, *p* < .001). Post hoc pairwise comparisons demonstrated that preoperative peritoneal to systemic difference had significantly higher values in horses with strangulating (mean [lower 95% CI‐upper 95% CI]; 3.12 [1.84–4.41]) and compared to non‐strangulating lesions (0.34 [−0.16–0.84], *p* = .001; Table S5). Horses with non‐strangulating lesions had significantly higher postoperative values at 24 h (2.65 [1.53–3.76], *p* = .007), 72 h (1.97 [1.20–2.75], *p* = 0.02), and 168 h (1.43 [0.63–2.24], *p* = .03) compared to preoperative values for the difference. Strangulating lesions had significantly lower postoperative values at 72 h (1.38 [0.94–1.82], *p* = .03) compared to preoperative values. No significant differences were present postoperatively between horses with strangulating and non‐strangulating lesions (Table S5).

For peritoneal total protein, the mixed model analysis revealed a significant main effect for time (F [2.5, 53.1] = 25.8, *p* < .001) and the interaction between time and lesion (F [3, 63] = 4.4, *p* = .007). Post hoc pairwise comparisons demonstrated that preoperative peritoneal total protein was significantly higher in strangulating (mean [lower 95% CI‐upper 95% CI]; 2.82 g/dL [1.99–3.55], *p* = .02) compared to non‐strangulating (1.67 g/dL [1.09–2.24]) lesions (Figure 1E; Tables S4 and S5). In addition, all postoperative concentrations in horses with non‐strangulating lesions were significantly higher than preoperative concentrations: 24 h (3.52 g/dL [2.81–4.13], *p* = .003), 72 h (4.78 g/dL [3.98–5.48], *p* < .001), and 168 h (4.34 g/dL [3.56–5.13], *p* = .002). No significant differences were present over time in horses with strangulating lesions, or postoperatively between horses with strangulating and non‐strangulating lesions.

Of the samples for which description of color was reported, peritoneal fluid color was serosanguineous in 18/24 (75%) preoperative samples and in 37/38 (97%) samples collected postoperatively. Of the postoperative samples collected, 38/73 (52%) of abdominocenteses yielded a peritoneal fluid volume >0.5 mL, but this volume could only be collected at all three postoperative time points in 3/26 (12%) horses. For the TNCC analysis, the mixed model revealed a significant main effect for time (F [1, 38] = 45.05, *p* < .001) only. Post hoc pairwise comparisons demonstrated that peritoneal TNCC was significantly increased at 24 h postoperatively (geometric mean [lower 95% CI‐upper 95% CI]; 15 996 cells/μL [3331–76 642] and 103 039 cells/μL [52 053–203 996]) compared to preoperative (1374 cells/μL [412–4587] and 1429 cells/μL [764–2675]) concentrations for the strangulating (*p* = .001) and non‐strangulating (*p* < .001) groups, respectively (Table 1 and Table S5). However, the residual variance was >99% for the model, indicating that most of the variability was likely due to individual fluctuations in TNCC within the horses over time. There were multiple missing data points for TNCC samples in the 72‐ and 168‐h time periods (Table 1). Of the eight horses that had samples available at 24 and 168 h postoperatively, the peritoneal TNCC decreased in all eight. Of the 12 available samples at 168 h postoperatively, peritoneal TNCC remained elevated above the reference range in five. There was no statistical correlation between TNCC and peritoneal lactate (*p* = .517) or total protein (*p* = .175).

**TABLE 1:** Peritoneal TNCC/μL values and neutrophil percentage in horses preoperatively and at 24, 72, and 168 h postoperatively or discharge.

Horse	Preoperatively		24‐h postoperatively		72‐h postoperatively		168‐h postoperatively (or discharge)	
	TNCC/μL	% Neut	TNCC/μL	% Neut	TNCC/μL	% Neut	TNCC/μL	% Neut
Horse 1	620	80%	50 830	95%	‐	62%	530	40%
Horse 4^a^	1370	57%	54 000	86%	‐	‐	380	60%
Horse 6	8020	66%	‐	‐	2350	55%	‐	‐
Horse 8^a^	1340	50%	46 060	80%	23 290	84%	‐	‐
Horse 9	620	30%	312 620	85%	‐	‐	‐	‐
Horse 17	3420	68%	93 790	76%	4130	83%	1800	71%
Horse 18	3880	29%	396 370	82%	‐	‐	‐	80%
Horse 20	4850	79%	82 430	92%	42 590	83%	26 440	80%
Horse 21	400	13%	104 970	70%	31 440	53%	‐	63%
Horse 22	630	79%	‐^b^	‐^b^	‐	‐	‐	‐
Horse 23	590	69%	‐^b^	‐^b^	‐^b^	81%^b^	88 410	74%
Horse 24	1340	32%	‐	‐	‐	48%	150	48%
Horse 2	6550	48%	‐	80%	‐	68%	15 440	63%
Horse 3	1280	46%	273 690	76%	‐	82%	46 550	80%
Horse 5	160	75%	‐^b^	‐^b^	‐	‐	‐	‐
Horse 7	52 190	91%	‐	‐	‐	‐	‐	71%
Horse 10	5670	86%	‐	94%	‐	87%	62 760	84%
Horse 11	500	59%	119 120	85%	‐	38%	700	56%
Horse 12^a^	‐	‐	1210	92%	‐	‐	‐	‐
Horse 13	4200	56%	49 790	81%	150	58%	‐	‐
Horse 14	370	77%	‐	74%	200	32%	‐	25%
Horse 15	1660	60%	700	74%	3910	65%	‐	20%
Horse 16	16 060	89%	7570	91%	‐	82%	‐	93%
Horse 19	610	62%	6810	88%	‐	72%	‐	‐
Horse 25	310	45%	13 880	96%	‐	67%	150	55%
Horse 26^a^	40	97%	69 000	91%	20 790	79%	7050	59%

*Note*: Horses shaded in gray had non‐strangulating lesions and horses in unshaded boxes had strangulating lesions.

Abbreviations: neut, neutrophil; TNCC, total nucleated cell count.

^a^ The horse did not survive to discharge.

^b^ Enterocentesis was inadvertently performed.

For the percentage of peritoneal neutrophils measured on cytology, the mixed model analysis revealed a significant main effect for time (F [1.8, 29.7] = 10.4, *p* < .001) only. Similar to TNCC, post hoc pairwise comparisons demonstrated that peritoneal percent neutrophils were significantly increased at 24 h postoperatively (mean [lower 95% CI‐upper 95% CI]; 85% [79–91%] and 84% [78–89%]) compared to preoperative (57% [43–71%] and 68% [57–79%]) concentrations for the strangulating (*p* = .009) and non‐strangulating (*p* = .01) groups, respectively (Table 1 and Table S5). At 24 h postoperatively, the percent neutrophils were above 80% in 13/20 available samples, but at 168 h postoperatively, the percent neutrophils were above 80% in only 2/18 available samples.

Four enterocenteses inadvertently occurred during this project out of a total of 73 postoperative abdominocenteses performed (5%). One horse (Horse 26) underwent an enterocentesis during diagnostic testing prior to enrollment in the study; therefore, a peritoneal fluid sample was collected at surgery for this horse. One horse (Horse 22) was removed from the study by the owner after an enterocentesis occurred at the 24‐h postoperative peritoneal fluid collection. Horses were treated according to clinician preference and no further complications related to the enterocentesis, such as peritonitis or cellulitis, were reported in any of the cases. Examining the peritoneal fluid concentrations after an enterocentesis did not reveal any obvious signs of further inflammation (Table 1 and Tables S2–S4). No other complications directly associated with the abdominocentesis procedure were reported during the study.

Of the enrolled horses, 22/26 (85%) survived to hospital discharge. Two horses (Horses 4 and 8) underwent a repeat exploratory laparotomy at 5 days and 32 h postoperatively, respectively, and were euthanized intraoperatively due to recurrence of the initial presenting lesion. Two horses were euthanized postoperatively (Horses 12 and 26) at 30 h and 10 days postoperatively, respectively, due to recurrent colic and persistent gastric reflux (Horse 26). A post‐mortem examination was performed on both horses, with Horse 12 having evidence of postoperative ileus, and Horse 26 having an adhesion just orad to the jejunoileostomy, preventing passage of ingesta. Due to low numbers of non‐survivors, differences between survivors and non‐survivors were not discernable. Of the surviving horses, two cases (Horse 1 and Horse 10) had sample collection prior to the final timepoint of the study, at 124 h and 132 h postoperatively, respectively.

Follow‐up was obtained for 17 (77%) of the 22 horses discharged. A total of 16 horses had follow‐up obtained at a minimum of 5 months postoperatively, while one additional horse (Horse 17) was euthanized at the University 3 months postoperatively due to recurrent colic, and follow‐up was obtained at that time. Of these 17 horses, three (18%) exhibited incisional drainage or infection, that had resolved by the time of follow‐up. One horse (6%) developed an incisional hernia, while another horse (6%) had an incisional hernia and incisional infection.

## 4 DISCUSSION

To the authors' knowledge, this is the first study reporting peritoneal lactate, TP, and TNCC values following exploratory laparotomy in horses with naturally‐occurring gastrointestinal disease. This information can serve as pilot data for larger studies to further investigate the changes that occur postoperatively in clinical cases.

In horses with naturally‐occurring colic, previous studies have utilized a single, or serial measurement, of peritoneal lactate to help determine which horses required an exploratory laparotomy.^4^, ^5^, ^7^, ^8^, ^9^, ^10^ Because lactate is a byproduct of anaerobic cellular metabolism, circulatory compromise and ischemia of the viscera can lead to higher than normal concentrations of lactate in the peritoneal fluid.^3^ In addition, many studies have demonstrated that strangulating obstructions have greater peritoneal lactate concentrations than non‐strangulating obstructions in horses with naturally‐occurring colic signs.^4^, ^5^, ^8^, ^10^ Preoperative peritoneal lactate concentrations in the current study supported this with strangulating lesions having higher preoperative peritoneal fluid lactate levels than horses with non‐strangulating lesions. Considering preoperative elevation, we hypothesized that peritoneal fluid lactate concentrations would decrease within the first postoperative week after lesion correction. This was only partially supported because concentrations decreased, on average, in the strangulating group over time, but not in the non‐strangulating group, with neither returning to normal levels within the study period. Since horses with strangulating lesions had higher preoperative peritoneal fluid lactate levels than horses with non‐strangulating lesions, a greater decrease may be possible after reperfusion of the gastrointestinal tract, which might account for different responses between groups. Additionally, resection of devitalized bowel in some strangulating cases may have contributed to a decreased peritoneal lactate in the postoperative period. It has been stated the lactate levels will decrease with fluid therapy,^6^, ^18^ but no data was presented in those studies to support the statement. Nonetheless, in most of the horses in this study, peritoneal lactate had not returned to normal after a week. It is important to note that in the postoperative period, peritoneal lactate concentrations are not different over time between strangulating and non‐strangulating groups, making it important to consider serial peritoneal lactates^5^ in the postoperative period as well. Serial lactate measurements could help discern if the concentration is increasing over time. Further research examining more horses for a longer postoperative period would be required to determine when the peritoneal lactate returns to normal and if serial increases in concentrations are predictive of recurrence or outcome.

Previous studies have shown that systemic lactate concentrations tend to decrease postoperatively following exploratory laparotomy^19^, ^20^, ^21^ and are often within normal limits within 24 h.^19^ This is similar to our study, where systemic lactate in most horses returned to normal by 24 h postoperatively; whereas, peritoneal fluid lactate remained elevated above the reference range in the majority of cases, supporting our hypothesis that systemic lactate normalizes more rapidly than peritoneal fluid lactate. In the postoperative patient, peritoneal fluid lactate decreases may be slower than systemic lactate decreases due to ongoing intra‐abdominal inflammation caused by surgical manipulation^12^, ^13^, ^22^, ^23^ and likely the underlying cause of the colic. Despite these observations, the rate and mechanism of peritoneal lactate clearance in horses has not been well established and may lag behind systemic clearance.

Previous literature has suggested that a peritoneal to systemic lactate ratio of >2 is indicative of a strangulating lesion^9^ and that the peritoneal to systemic lactate difference could discriminate strangulating lesions from non‐strangulating lesions preoperatively.^7^, ^8^ Our current study supported these findings in the preoperative samples. However, in the postoperative period, usefulness of the ratio and difference was not evident. On average, both the ratio and difference similarly increased postoperatively in the non‐strangulating group, with no difference between the non‐strangulating and strangulating lesion group. On average, the ratio was above 2 for both groups for all postoperative time periods, meaning that in the postoperative period, a ratio of 2 is more indicative of a typical postoperative response. Further research in a larger group of horses is needed to delineate the usefulness of these values in the postoperative period.

At the preoperative time point, peritoneal TP was significantly higher in horses in our study with strangulating lesions compared to non‐strangulating lesions, which is in line with previously published findings.^8^ Our hypothesis that peritoneal TP would decrease postoperatively was not supported. Peritoneal TP increased postoperatively and remained elevated throughout the study in the non‐strangulating group, similar to other reports that found peritoneal TP remained elevated for up to 14 days after abdominal surgery in healthy horses and ponies.^12^, ^13^, ^22^ This is likely due to peritoneal inflammation following laparotomy and surgical manipulation. Additionally, peritoneal fluid was subjectively difficult to collect, which is similar to a previous study that attempted to retrieve peritoneal fluid in healthy horses following an exploratory laparotomy.^23^ It is likely that this is due to resolution of inflammation leading to decreased free fluid within the abdomen. Peritoneal fluid samples were collected in glass tubes containing EDTA, which has previously been shown to increase TP levels when not filled appropriately.^24^ It is possible that this is another reason for the increase in TP postoperatively due to several samples with low fluid volume.

A previous study has shown that in healthy horses, normal peritoneal fluid is pale yellow in color and serosanguineous fluid can indicate a strangulating lesion.^2^ However, all but one of the postoperative samples with color recorded in our study were serosanguineous, indicating that the color of peritoneal fluid is not a reliable indicator for lesion type in the immediate postoperative period. It is important to note that color was only recorded in the current study by the pathologist who analyzed the fluid, meaning that about half the samples did not have a reported color. Nonetheless, our results are supported by a previous study that found peritoneal fluid to be red in the first 3 days postoperatively in healthy horses undergoing a small colon resection and anastomosis.^13^ It is possible that the color remained serosanguineous longer in our study because the study population only included those with naturally‐occurring disease. This could also increase the chance for contamination of the sample from peripheral blood. A previous study has shown that up to 17% peripheral blood contamination does not impact the total protein of peritoneal fluid samples,^25^ but it is also possible that a higher percent of peripheral blood contamination occurred during collection of some postoperative samples.

An inability to collect 0.5 mL of fluid led to a large number of missing data points regarding peritoneal TNCC and percent neutrophils. However, this information is still valuable as pilot data. The peritoneal TNCC results were similar to a previous study in healthy horses where peritoneal TNCC peaked 24 h after surgery and then declined, but remained above normal reference values for 7 days postoperatively.^13^ This is likely due to postoperative inflammation from the initial disease process and surgical manipulation. The TNCC had not reliably returned to the reference range by 7 days postoperatively, indicating there is still ongoing inflammation present at that point postoperatively. The percent neutrophils followed a similar pattern, with values increasing 24 h postoperatively and then trending towards normal by the end of the study period.

In a previous study, peritoneal lactate and TP were found to be significantly higher preoperatively in horses with small intestinal lesions compared to large intestinal lesions.^26^ Even though most of the strangulating lesions in the current study were small intestinal, and most of the non‐strangulating lesions were large intestinal, it is unlikely that the anatomic site had any specific effect on the peritoneal fluid values, as there were no postoperative statistical differences between lesion groups in any of the parameters measured. However, more horses would need to be studied to determine this. The majority of cases enrolled in this study had at least one segment of bowel opened during surgery. Due to the small number of horses that did not have bowel opened, it is difficult to determine if this caused any variation in results. However, it is likely that opening the bowel in a controlled manner did not have a large impact on measured values within the peritoneal fluid. In a previous report, resection and anastomosis of the small colon in healthy horses did not cause a significantly different inflammatory response within peritoneal fluid compared to manipulation of the intestine alone.^13^ Carboxymethylcellulose was applied during the majority of cases as well. Previous work found that there was no negative effect on wound healing or clinical outcome when intraperitoneal carboxymethylcellulose was used, but its effect on the characteristics of peritoneal fluid is unknown.^27^ It is possible that carboxymethylcellulose could have an effect on peritoneal fluid characteristics postoperatively, and as such provides an area for further study. Abdominal lavage was performed at the end of surgery in only a few horses in this study, but it is unlikely to affect the peritoneal fluid based on previous work where lavage did not affect the inflammatory response of the postoperative peritoneal fluid after a small colon resection/anastomosis in normal horses^13^; nonetheless, this is another area that warrants further study.

Inadvertent enterocentesis occurred in 5% of abdominocenteses performed in this study, which is similar to previously reported rates of 4%–5%.^2^ While enterocentesis has been reported to increase TNCC in peritoneal fluid in healthy horses,^28^ this was not evident in the current study. It is possible that this was not detectable because of a laparotomy being performed on horses with naturally‐occurring disease. Similar to a previous study,^2^ no further complications were noted after enterocentesis occurred. Incisional infections, drainage, and hernias are all known postoperative complications of exploratory laparotomy in horses.^11^, ^29^, ^30^, ^31^ The rate of incisional drainage and hernia formation in this study was similar to these previously reported ranges. Therefore, in this small sample size, serial postoperative abdominocenteses do not appear to increase the risk of these complications.

The major limitation of the present study is that it included a small sample size with multiple different gastrointestinal lesions. While we had hoped to find clear patterns within the studied characteristics of peritoneal fluid indicating the need for a second laparotomy or euthanasia, our sample size was too small to find evidence to support such a claim. No normal control horses were used because that information is already present in the literature. This study had several additional limitations. Cases were managed by multiple clinicians, which introduced variability. Fluid therapy, which could influence lactate levels, was given according to clinician discretion in the pre‐ and postoperative periods and was not standardized. Sample collection preoperatively was not always possible, necessitating collection intraoperatively in some cases. This methodology has previously been used when preoperative sample collection was not achievable.^32^ The study period ended at 7 days postoperatively, which was prior to peritoneal fluid values returning to normal in many horses. However, this is similar in duration to previous studies evaluating postoperative peritoneal fluid.^12^, ^13^ Additionally, extending hospitalization solely to monitor peritoneal fluid in an otherwise healthy postoperative patient is impractical and not necessarily in the best interest of the patient. Due to the clinical nature of the study, there were missing data points. Follow up was collected via phone call or email with the owner and a recheck examination by a veterinarian was not routinely performed.

In conclusion, this study reports serial peritoneal and systemic lactate, as well as peritoneal TP and TNCC values in horses with naturally‐occurring gastrointestinal disease following exploratory laparotomy. This information can serve as pilot data for larger studies to further investigate the changes that occur postoperatively in clinical cases. Additionally, the postoperative values reported vary from reference intervals previously published for preoperative peritoneal fluid; therefore, those reference values should not be utilized in the postoperative period until further studies have been performed. Further work on this topic will increase the utility of abdominocentesis postoperatively with the goal of giving clinicians another diagnostic tool in the management of challenging cases experiencing postoperative colic.

## AUTHOR CONTRIBUTIONS

Granello M, DVM: Conception and design of the work, collection and interpretation of the data, drafting and revision of the manuscript. Young J, DVM, DACVS (Large Animal): Conception and design of the work, collection and interpretation of the data, drafting and revision of the manuscript. Cleff D, BVetMed: Data acquisition and revision of the manuscript. Banks E, BS: Data acquisition and revision of the manuscript. Trumble T, DVM, PhD, DACVS: Conception and design of the work, analysis and interpretation of the data, drafting and revision of the manuscript. All authors provided a critical review of the manuscript and endorse the final version. All authors are aware of their respective contributions and have confidence in the integrity of all contributions.

## FUNDING INFORMATION

This project was supported by funding from the ACVS Foundation Diplomate Research Grant and by the University of Minnesota College of Veterinary Medicine Internal Grants funding program.

## CONFLICT OF INTEREST STATEMENT

The authors declare no conflicts of interest related to this report.

## Supporting information


**Data S1:** Supporting Information.
