# Supplementary material for: Serial postoperative peritoneal fluid analyses in horses with naturally‐occurring strangulating and non‐strangulating gastrointestinal lesions
Source: Vet Surg. 2026 Feb 22;55(3):568–79. doi: 10.1111/vsu.70089 (PMC13069231; doi:10.1111/vsu.70089)
Supplement: Supplementary file 1 — Data S1: Supporting Information. [file VSU-55-568-s001.docx]

| **Horse** | **Age (yrs)** | **Breed** | **Sex** | **Wt (kg)** | **Surgery collected pre-op peritoneal sample** | **Strang or non-strang** | **Surgical diagnosis** | **Resection** | **Enterotomy (pelvic flexure, unless specified)** | **CMC used** | **Abdominal lavage performed prior to closure** | **Surgeon** | **Survival to discharge** |
| --- | --- | --- | --- | --- | --- | --- | --- | --- | --- | --- | --- | --- | --- |
| **1** | 11 | TB | MC | 541 | Yes | Non | Large colon nephrosplenic entrapment | No | No | Yes | No | 1 | Yes |
| **2** | 7 | TB | MC | 533 | Yes | S | 360 large colon volvulus and sand impaction | No | Yes | Yes | Yes with 2L saline | 2 | Yes |
| **3** | 18 | SB | F | 472 | Yes | S | Strangulating lipoma aboral jejunum (3 feet) | Yes (j-j) | No | Yes | No | 2 | Yes |
| **4** | 23 | Pony | MC | 182 | No | Non | Small colon fecalith and large colon impaction | No | Yes | No | No | 3 | No |
| **5** | 13 | CP | MC | 431 | Yes | S | Strangulating lipoma mid-jejunum (2 feet) | Yes (j-j) | No | Yes | No | 3 | Yes |
| **6** | 19 | QH | MC | 624 | Yes | Non | Transverse colon fecalith | No | Yes | No | Yes with 5L LRS | 3 | Yes |
| **7** | 24 | QH | MC | 481 | Yes | S | Strangulating lipoma mid-jejunum (1 foot) | Yes (j-j) | No | Yes | No | 1 | Yes |
| **8** | 18 | SB | MC | 501 | No | Non | Type I cecal impaction and large colon displacement | No | Yes (typhlotomy) | No | Yes with 10L LRS | 3 | No |
| **9** | 22 | KWPN | MC | 686 | Yes | Non | Large colon right dorsal displacement and impaction | No | Yes | No | No | 3 | Yes |
| **10** | 13 | WP | MC | 286 | Yes | S | Strangulation of oral ileum (1 foot) and aboral jejunum (1 foot) and large colon impaction | Yes (j-i) | Yes | Yes | No | 1 | Yes |
| **11** | 17 | QH | MC | 516 | Yes | S | Strangulating lipoma jejunum (15 feet) | Yes (j-j) | Yes | Yes | No | 1 | Yes |
| **12** | 21 | LUS | MC | 568 | Yes | S | 360 degree large colon volvulus, gastric impaction, small intestinal wall mass | No | Yes (PF and jejunum) | Yes | No | 3 | No |
| **13** | 17 | Arab | MC | 427 | No | S | Strangulating lipoma distal jejunum (6 inches) | Yes (j-i) | No | Yes | No | 3 | Yes |
| **14** | 15 | APH | MC | 600 | No | S | Strangulating lipoma distal jejunum and ileum | No | No | Yes | No | 3 | Yes |
| **15** | 15 | APH | MC | 509 | Yes | S | Epiploic foramen entrapment of distal jejunum and ileum (2 feet) | Yes (j-i) | No | No | No | 4 | Yes |
| **16** | 21 | Must | MC | 571 | Yes | S | Strangulating lipoma aboral jejunum and oral ileum (24 feet); hemoabdomen | Yes (j-i) | No | Yes | No | 3 | Yes |
| **17** | 16 | SDH | F | 563 | No | Non | Large colon right dorsal displacement and impaction | No | Yes | Yes | No | 3 | Yes |
| **18** | 11 | Pony | MC | 186 | Yes | Non | Small colon fecalith and large colon impaction | No | Yes | Yes | No | 5 | Yes |
| **19** | 10 | Quarab | MC | 509 | No | S | Mesenteric volvulus small intestine | No | Yes | Yes | No | 4 | Yes |
| **20** | 3 | GVH | F | 404 | Yes | Non | Large colon right dorsal displacement and impaction | No | Yes | Yes | No | 5 | Yes |
| **21** | 9 | SF | MC | 655 | Yes | Non | Large colon right dorsal displacement | No | No | Yes | No | 5 | Yes |
| **22** | 25 | TWH | MC | 523 | No | Non | Large colon right dorsal displacement and impaction | No | Yes | Yes | No | 5 | Yes |
| **23** | 9 | TB | F | 481 | Yes | Non | Large colon right dorsal displacement and cecal tympany | No | No | No | No | 3 | Yes |
| **24** | 15 | SP | MC | 166 | No | Non | Large colon impaction | No | Yes | Yes | No | 3 | Yes |
| **25** | 14 | QH | MC | 643 | No | S | Strangulating lipoma aboral jejunum and oral ileum (15 feet) | Yes (j-i) | Yes | Yes | No | 3 | Yes |
| **26** | 14 | TB | MC | 566 | Yes | S | Strangulating lipoma aboral jejunum and oral ileum (4 feet) | Yes (j-i) | No | Yes | No | 3 | No |

**Supplementary Table 1**: Signalment and surgical information for enrolled cases.

yrs=years; TB=Thoroughbred; SB=Saddlebred; CP=Connemara Pony; QH=Quarter Horse; KWPN=Dutch Warmblood; WP=Welsh Pony; LUS=Lusitano; APH=American Paint Horse; Must=Mustang; SDH=Spotted Draft Horse; GVH=Gypsy Vanner Horse; SF=Selle Francais; TWH=Tennessee Walking Horse; SP=Shetland Pony; Quarab=Quarter Horse Arabian cross; MC=Male castrated; F=Female; Wt=weight; Strang or S=strangulating; non-strang or Non=non-strangulating; j-j=jejunojejunostomy; j-i=jejunoileostomy; PF=pelvic flexure; CMC=carboxymethylcellulose

| **Horse** | **Pre-op peritoneal lactate**  **(mmol/L)** | **24 hour post-op peritoneal lactate**  **(mmol/L)** | **72 hour post-op peritoneal lactate**  **(mmol/L)** | **168 hour post-op (or discharge) peritoneal lactate**  **(mmol/L)** |
| --- | --- | --- | --- | --- |
| Horse 1 | 1.3 | 1.7 | 1.7 | 1.1 |
| Horse 4* | 4.8 | 2.8 | 4.8 | 3.2 |
| Horse 6 | 0.9 | 2.9 | 1.5 | 2.7 |
| Horse 8* | 2.9 | 3.2 | 5.2 | - |
| Horse 9 | 2.7 | 3.6 | 2.5 | 2.0 |
| Horse 17 | 1.0 | 8.4 | 4.9 | 4.8 |
| Horse 18 | 2.5 | 2.8 | 2.2 | 2.2 |
| Horse 20 | 2.2 | 2.6 | 2.4 | 2.1 |
| Horse 21 | 1.4 | 2.1 | 1.7 | 1.9 |
| Horse 22 | 1.8 | -^ | - | - |
| Horse 23 | 1.5 | 3.3^ | 1.9^ | 2.3 |
| Horse 24 | 2.7 | 5.2 | 3.5 | 1.9 |
| Horse 2 | 2.0 | 3.1 | 1.5 | 1.1 |
| Horse 3 | 3.8 | 2.7 | 1.3 | 3.6 |
| Horse 5 | 3.2 | -^ | 1.1 | 0.8 |
| Horse 7 | 7.8 | 1.2 | 1.0 | 1.0 |
| Horse 10 | 3.6 | 5.2 | 3.2 | 1.7 |
| Horse 11 | 5.5 | 2.5 | 1.2 | 2.1 |
| Horse 12 * | 5.1 | 4.8 | 5.4 | - |
| Horse 13 | 2.5 | 1.6 | 1.9 | 1.4 |
| Horse 14 | 4.3 | 2.8 | 2.6 | 2.4 |
| Horse 15 | 2.8 | 1.9 | 2.0 | 3.6 |
| Horse 16 | 14.5 | 5.1 | 2.7 | 4.4 |
| Horse 19 | 4.2 | 3.8 | 2.5 | - |
| Horse 25 | 3.5 | 2.3 | 1.8 | 1.3 |
| Horse 26* | 8.2 | 5.3 | 3.2 | 1.8 |

**Supplemental Table 2**: Peritoneal lactate concentrations in horses pre-operatively (pre-op) and at 24, 72, and 168 hours post-operatively (post-op) or discharge. *= horse did not survive to discharge. ^ = horse experienced inadvertent enterocentesis at this time point. Horses shaded in gray had non-strangulating lesions and horses in unshaded boxes had strangulating lesions.

| **Horse** | **Pre-op systemic lactate (mmol/L)** | **24 hour post-op systemic lactate (mmol/L)** | **72 hour post-op systemic lactate (mmol/L)** | **168 hour post-op (or discharge) systemic lactate (mmol/L)** |
| --- | --- | --- | --- | --- |
| Horse 1 | 1.7 | 0.5 | 0.3 | 0.4 |
| Horse 4* | 3.4 | 1.1 | 0.9 | - |
| Horse 6 | 0.5 | 0.7 | 0.9 | 1.0 |
| Horse 8* | 1.7 | 0.8 | 2.9 | - |
| Horse 9 | 2.2 | 0.6 | 0.6 | 0.6 |
| Horse 17 | 1.0 | 1.5 | 0.8 | 0.8 |
| Horse 18 | 2.7 | 0.8 | 1.0 | 1.6 |
| Horse 20 | 2.3 | 0.9 | 0.8 | 1.0 |
| Horse 21 | 0.5 | 0.6 | 0.9 | 0.7 |
| Horse 22 | 1.1 | 0.7^ | - | - |
| Horse 23 | 2.9 | 1.2^ | 0.5^ | 0.7 |
| Horse 24 | 1.7 | 0.8 | 1.0 | 1.3 |
| Horse 2 | 1.3 | 0.7 | 0.7 | 0.7 |
| Horse 3 | 1.9 | 0.8 | 0.8 | 1.0 |
| Horse 5 | 1.7 | 0.6^ | 0.6 | 0.9 |
| Horse 7 | 3.0 | 0.6 | 0.6 | 0.3 |
| Horse 10 | 2.0 | 0.9 | 0.8 | 0.9 |
| Horse 11 | 1.7 | 0.8 | 0.8 | 1.2 |
| Horse 12 * | 1.4 | 3.3 | 3.0 | - |
| Horse 13 | 1.7 | 0.6 | 0.6 | 0.7 |
| Horse 14 | 0.7 | 0.5 | 0.6 | 0.6 |
| Horse 15 | 1.3 | 0.6 | 0.6 | 0.8 |
| Horse 16 | 6.4 | 1.6 | 1.0 | 1.4 |
| Horse 19 | 1.5 | 1.0 | 0.5 | - |
| Horse 25 | 1.4 | 0.8 | 0.5 | 0.8 |
| Horse 26* | 1.3 | 2.6 | 1.0 | 1.0 |

**Supplemental Table 3**: Systemic lactate concentrations in horses pre-operatively (pre-op) and at 24, 72, and 168 hours post-operatively (post-op) or discharge. *= horse did not survive to discharge. ^ = horse experienced inadvertent enterocentesis at this time point. Horses shaded in gray had non-strangulating lesions and horses in unshaded boxes had strangulating lesions.

| **Horse** | **Pre-op peritoneal TP (g/dL)** | **24 hour post-op peritoneal TP (g/dL)** | **72 hour post-op peritoneal TP (g/dL)** | **168 hour post-op (or discharge) peritoneal TP (g/dL)** |
| --- | --- | --- | --- | --- |
| Horse 1 | 1.3 | 4.8 | 5.1 | 4.4 |
| Horse 4* | 3.1 | 2.8 | 3.0 | 2.0 |
| Horse 6 | 1.5 | 5.2 | 5.9 | 4.3 |
| Horse 8* | 1.6 | 2.5 | 3.3 | - |
| Horse 9 | 2.0 | 2.9 | 6.6 | 5.8 |
| Horse 17 | 1.6 | 3.2 | 4.8 | 5.5 |
| Horse 18 | 1.0 | 4.6 | 6.0 | 5.2 |
| Horse 20 | 2.3 | 3.7 | 4.6 | 3.9 |
| Horse 21 | 0.5 | 3.4 | 4.5 | 4.6 |
| Horse 22 | 3.4 | -^ | - | - |
| Horse 23 | 0.6 | 2.3^ | 4.1^ | 3.4 |
| Horse 24 | 1.1 | 2.8 | 4.1 | 4.3 |
| Horse 2 | 1.3 | 4.1 | 5.0 | 5.0 |
| Horse 3 | - | 4.0 | 6.6 | 4.2 |
| Horse 5 | 2.6 | -^ | 2.1 | - |
| Horse 7 | 3.8 | 6.3 | 4.3 | 6.0 |
| Horse 10 | 3.3 | 3.5 | 4.2 | 3.5 |
| Horse 11 | 4.4 | 2.7 | 5.8 | 4.2 |
| Horse 12 * | 1.0 | 1.5 | 1.1 | - |
| Horse 13 | 1.2 | 2.9 | 3.0 | 4.5 |
| Horse 14 | 2.1 | 2.8 | 4.2 | 3.9 |
| Horse 15 | 3.0 | 3.0 | 4.4 | 4.7 |
| Horse 16 | 4.3 | 2.1 | 4.3 | 2.8 |
| Horse 19 | 1.2 | 3.0 | 4.8 | - |
| Horse 25 | 3.4 | 2.7 | 3.0 | 4.3 |
| Horse 26* | 4.4 | 3.6 | 4.0 | 4.1 |

**Supplemental Table 4**: Peritoneal total protein (TP) concentrations in horses pre-operatively (pre-op) and at 24, 72, and 168 hours post-operatively (post-op) or discharge. *= horse did not survive to discharge. ^ = horse experienced inadvertent enterocentesis at this time point. Horses shaded in gray had non-strangulating lesions and horses in unshaded boxes had strangulating lesions.

**Supplemental Table 5**: Tables of the results for the mixed model analysis performed. Each table is headed by the dependent variable analyzed. P<0.05 was considered significant for all analyses.

**Peritoneal Lactate**

*Log transformed mixed model results*:

***Fixed Effects F(DFn DFd) P value Variance % Variance***

*Time* F(2.5, 54.4) = 5.148 0.005 Horse (between): 0.017 40%

*Lesion*  F(1, 24) = 0.188 0.669 Residual (within): 0.026 60%

*Time x Lesion* F(3, 65) = 11.720 <0.0001

**Geisser-Greenhouse's Ɛ**: 0.8361

Back transformed (10^x) geometric means (median) with multiple pairwise comparisons (Tukey’s):

|  | ***Time (hours)*** | | | | | | | |
| --- | --- | --- | --- | --- | --- | --- | --- | --- |
| ***Lesion*** | **0** | | **24** | | **72** | | **168** | |
| *Strangulating* | n=14 4.395^a^ | | n=13 2.858^a,c^ | | n=14 2.004^b^ | | n=12 1.901^b,c^ | |
| *Non-Strangulating* | n=12 1.923^a,b^ | | n=11 3.184^a^ | | n=11 2.649^a,b^ | | n=10 2.312^b^ | |
| *95% CI of difference* | 1.517 | 3.443 | 0.628 | 1.355 | 0.509 | 1.119 | 0.533 | 1.225 |
| *Adj P value* | 0.0003 | | 0.671 | | 0.153 | | 0.299 | |

Adjusted (Adj) P Values denote differences between Strangulating and Non-Strangulating horses for that given time point.

Like letters represent no significant difference between time points within a given lesion within a row.

**Systemic Lactate**

*Log transformed mixed model results*:

***Fixed Effects F(DFn DFd) P value Variance % Variance***

*Time* F(2.2, 49.5) = 16.36 <0.0001 Horse (between): 0.013 28%

*Lesion*  F(1, 24) = 0.033 0.858 Residual (within): 0.033 72%

*Time x Lesion* F(3, 66) = 0.331 0.803

**Geisser-Greenhouse's Ɛ**: 0.7496

Back transformed (10^x) geometric means (median) with multiple pairwise comparisons (Tukey’s):

|  | ***Time (hours)*** | | | | | | | |
| --- | --- | --- | --- | --- | --- | --- | --- | --- |
| ***Lesion*** | **0** | | **24** | | **72** | | **168** | |
| *Strangulating* | n=14 1.694^a^ | | n=14 0.914^b^ | | n=14 0.759^b^ | | n=12 0.832^b^ | |
| *Non-Strangulating* | n=12 1.545^a^ | | n=12 0.811^b^ | | n=11 0.817^a,b^ | | n=9 0.869^a,b^ | |
| *95% CI of difference* | 0.685 | 1.758 | 0.780 | 1.637 | 0.597 | 1.413 | 0.662 | 1.413 |
| *Adj P value* | 0.683 | | 0.504 | | 0.686 | | 0.857 | |

Adjusted (Adj) P Values denote differences between Strangulating and Non-Strangulating horses for that given time point.

Like letters represent no significant difference between time points within a given Lesion within a row.

**Peritoneal to Systemic Lactate Ratio**

*Log transformed mixed model results*:

***Fixed Effects F(DFn DFd) P value Variance % Variance***

*Time* F(2.7, 57.5) = 11.880 <0.0001 Horse (between): 0.003 9%

*Lesion*  F(1, 24) = 0.181 0.675 Residual (within): 0.031 91%

*Time x Lesion* F(3, 64) = 8.310 <0.0001

**Geisser-Greenhouse's Ɛ**: 0.8976

Back transformed (10^x) geometric means (median) with multiple pairwise comparisons (Tukey’s):

|  | ***Time (hours)*** | | | | | | | |
| --- | --- | --- | --- | --- | --- | --- | --- | --- |
| ***Lesion*** | **0** | | **24** | | **72** | | **168** | |
| *Strangulating* | n=14 2.600^a^ | | n=13 3.097^a^ | | n=14 2.636^a^ | | n=12 2.275^a^ | |
| *Non-Strangulating* | n=12 1.233^a^ | | n=11 3.890^b^ | | n=11 3.221^b^ | | n=9 2.630^b^ | |
| *95% CI of difference* | 1.463 | 3.034 | 0.596 | 1.089 | 0.561 | 1.189 | 0.568 | 1.321 |
| *Adj P value* | 0.0003 | | 0.151 | | 0.275 | | 0.482 | |

Adjusted (Adj) P Values denote differences between Strangulating and Non-Strangulating horses for that given time point.

Like letters represent no significant difference between time points within a given Lesion within a row.

**Peritoneal to Systemic Lactate Difference**

*Cube root transformed mixed model results*:

***Fixed Effects F(DFn DFd) P value Variance % Variance***

*Time* F(1.9, 40.5) = 6.230 0.005 Horse (between): 0.039 23%

*Lesion*  F(1, 24) = 1.775 0.195 Residual (within): 0.127 77%

*Time x Lesion* F(3, 64) = 17.53 <0.0001

**Geisser-Greenhouse's Ɛ**: 0.6326

Back transformed (x^3) arithmetic means with multiple pairwise comparisons (Tukey’s):

|  | ***Time (hours)*** | | | | | | | |
| --- | --- | --- | --- | --- | --- | --- | --- | --- |
| ***Lesion*** | **0** | | **24** | | **72** | | **168** | |
| *Strangulating* | n=14 2.674^a^ | | n=13 1.847^a,b^ | | n=14 1.225^b^ | | n=12 0.818^a,b^ | |
| *Non-Strangulating* | n=12 0.041^a^ | | n=11 2.477^b^ | | n=11 1.829^b,c^ | | n=9 1.431^c^ | |
| *95% CI of difference* | 0.125 | 3.997 | -0.024 | 0.001 | -0.039 | 0.0002 | -0.128 | 0.007 |
| *Adj P value* | 0.001 | | 0.346 | | 0.149 | | 0.355 | |

Adjusted (Adj) P Values denote differences between Strangulating and Non-Strangulating horses for that given time point.

Like letters represent no significant difference between time points within a given Lesion within a row.

**Peritoneal Total Protein**

*No transformation mixed model results*:

***Fixed Effects F(DFn DFd) P value Variance % Variance***

*Time* F(2.5, 53.1) = 25.830 <0.0001 Horse (between): 0.396 30%

*Lesion*  F(1, 24) = 0.007 0.933 Residual (within): 0.909 70%

*Time x Lesion* F(3, 63) = 4.443 0.007

**Geisser-Greenhouse's Ɛ**: 0.8434

LS Means with multiple pairwise comparisons (Tukey’s):

|  | ***Time (hours)*** | | | | | | | |
| --- | --- | --- | --- | --- | --- | --- | --- | --- |
| ***Lesion*** | **0** | | **24** | | **72** | | **168** | |
| *Strangulating* | n=13 2.819^a,b^ | | n=13 3.207^a^ | | n=14 4.057^a,b^ | | n=11 4.115^b^ | |
| *Non-Strangulating* | n=12 1.667^a^ | | n=11 3.521^b^ | | n=11 4.775^c^ | | n=10 4.344^b,c^ | |
| *95% CI of difference* | 0.180 | 2.025 | -1.136 | 0.682 | -1.719 | 0.378 | -0.949 | 0.851 |
| *Adj P value* | 0.021 | | 0.610 | | 0.199 | | 0.910 | |

Adjusted (Adj) P Values denote differences between Strangulating and Non-Strangulating horses for that given time point.

Like letters represent no significant difference between time points within a given Lesion within a row.

**Peritoneal Total Nucleated Cell Count**

*Log transformed mixed model results for Time 0 and 24 hour samples only*:

***Fixed Effects F(DFn DFd) P value Variance % Variance***

*Time* F(1, 38) = 45.05 <0.0001 Horse (between): 0.0001 <1%

*Lesion*  F(1, 38) = 3.602 0.065 Residual (within): 0.479 >99%

*Time x Lesion* F(1, 38) = 3.310 0.077

**Geisser-Greenhouse's Ɛ**: Not applicable

Back transformed (10^x) geometric means (median) uncorrected Fisher’s LSD:

|  | ***Time (hours)*** | | | | | | | |
| --- | --- | --- | --- | --- | --- | --- | --- | --- |
| ***Lesion*** | **0** | | **24** | | **72*** | | **168*** | |
| *Strangulating* | n=13 1374.042^a^ | | n=9 15995.580^b^ | | n=4 NA | | n=6 NA | |
| *Non-Strangulating* | n=12 1428.894^a^ | | n=8 103038.612^b^ | | n=5 NA | | n=6 NA | |
| *95% CI of difference* | 0.875 | 3.496 | 0.032 | 0.743 | NA | NA | NA | NA |
| *Adj P value* | 0.951 | | 0.021 | | NA | | NA | |

* **Note that Time 72 and 168 were NOT analyzed due to the number of missing samples.**

Adjusted (Adj) P Values denote differences between Strangulating and Non-Strangulating horses for that given time point.

Like letters represent no significant difference between time points within a given Lesion within a row.

**Peritoneal % Neutrophils**

*No transformation mixed model results*:

***Fixed Effects F(DFn DFd) P value Variance % Variance***

*Time* F(1.8, 29.7) = 10.36 0.0006 Horse (between): 0.009 30%

*Lesion*  F(1, 24) = 0.094 0.762 Residual (within): 0.021 70%

*Time x Lesion* F(3, 50) = 1.185 0.325

**Geisser-Greenhouse's Ɛ**: 0.5934

LS Means with multiple pairwise comparisons (Tukey’s):

|  | ***Time (hours)*** | | | | | | | |
| --- | --- | --- | --- | --- | --- | --- | --- | --- |
| ***Lesion*** | **0** | | **24** | | **72** | | **168** | |
| *Strangulating* | n=14 0.567^a^ | | n=10 0.854^b,c^ | | n=10 0.692^a,c^ | | n=10 0.630^a^ | |
| *Non-Strangulating* | n=11 0.683^a^ | | n=10 0.844^b^ | | n=9 0.662^a,b^ | | n=8 0.616^a,b^ | |
| *95% CI of difference* | -0.283 | 0.054 | -0.058 | 0.094 | -0.012 | 0.214 | -0.022 | 0.247 |
| *Adj P value* | 0.174 | | 0.623 | | 0.566 | | 0.888 | |

Adjusted (Adj) P Values denote differences between Strangulating and Non-Strangulating horses for that given time point.

Like letters represent no significant difference between time points within a given Lesion within a row.
